# Contemporary Neuroprotection Strategies during Cardiac Surgery: State of the Art Review

**DOI:** 10.3390/ijerph182312747

**Published:** 2021-12-03

**Authors:** Palesa Motshabi-Chakane, Palesa Mogane, Jacob Moutlana, Gontse Leballo-Mothibi, Sithandiwe Dingezweni, Dineo Mpanya, Nqoba Tsabedze

**Affiliations:** 1 Department of Anaesthesiology, Faculty of Health Sciences, School of Clinical Medicine, University of the Witwatersrand, Johannesburg 2193, South Africa; Palesa.Motshabi@wits.ac.za (P.M.-C.); moganep@gmail.com (P.M.); drhlamatsi@gmail.com (J.M.); gleballomothibi@gmail.com (G.L.-M.); sdingezweni@ymail.com (S.D.); 2 Department of Internal Medicine, Division of Cardiology, Faculty of Health Sciences, School of Clinical Medicine, University of the Witwatersrand, Johannesburg 2193, South Africa; Dineo.Mpanya@wits.ac.za

**Keywords:** neuroprotection, cerebrovascular, delirium, cardiac surgery, cardiac anaesthesia

## Abstract

Open-heart surgery is the leading cause of neuronal injury in the perioperative state, with some patients complicating with cerebrovascular accidents and delirium. Neurological fallout places an immense burden on the psychological well-being of the person affected, their family, and the healthcare system. Several randomised control trials (RCTs) have attempted to identify therapeutic and interventional strategies that reduce the morbidity and mortality rate in patients that experience perioperative neurological complications. However, there is still no consensus on the best strategy that yields improved patient outcomes, such that standardised neuroprotection protocols do not exist in a significant number of anaesthesia departments. This review aims to discuss contemporary evidence for preventing and managing risk factors for neuronal injury, mechanisms of injury, and neuroprotection interventions that lead to improved patient outcomes. Furthermore, a summary of existing RCTs and large observational studies are examined to determine which strategies are supported by science and which lack definitive evidence. We have established that the overall evidence for pharmacological neuroprotection is weak. Most neuroprotective strategies are based on animal studies, which cannot be fully extrapolated to the human population, and there is still no consensus on the optimal neuroprotective strategies for patients undergoing cardiac surgery. Large multicenter studies using universal standardised neurological fallout definitions are still required to evaluate the beneficial effects of the existing neuroprotective techniques.

## 1. Introduction

Neuroprotection encompasses strategies that preserve neuronal structure and function. Neurological fallout must be prevented in all patients undergoing any form of surgery, particularly those referred for cardiac surgery. For example, in a study involving 10,250 patients that underwent cardiac surgery, 221 (2%) experienced a postoperative stroke, and the duration of hospitalisation was significantly longer in these patients compared to those without postoperative stroke (10 vs. 16 days, *p* < 0.001) [1]. Numerous randomised controlled trials (RCTs) have investigated the efficacy of pharmacological and non-pharmacological interventions that reduce neurological injury during and after cardiac surgery [2,3,4]. However, there is still controversy on the optimal neuroprotection strategy that leads to better patient outcomes.

This review aims to discuss contemporary evidence for preventing and managing risk factors for neuronal injury, mechanisms of injury, and neuroprotection interventions that lead to improved patient outcomes.

## 2. Materials and Methods

Data on RCTs using various neuroprotective agents was obtained after conducting a systematic literature search in PUBMED, Scopus and Google scholar. The following search string was used: (neuroprotection strategies OR neuroprotection OR pharmacological therapy OR non-pharmacological therapy) AND (cardiac surgery OR cardiopulmonary bypass surgery) AND (postoperative cognitive dysfunction OR stroke OR delirium OR seizure) AND adult patients. We included randomised control trials, systematic reviews and meta-analyses published in the past 15 years. The focus of the literature search was on pharmacological and non-pharmacological neuroprotective strategies (Table 1). Where systematic reviews or meta-analyses of the RCTs of interventions were available, these were preferentially reported on rather than the individual RCTs.

## 3. Incidence and Prevalence of Neurological Deficit after Cardiac Surgery

The International Code of Nomenclature recommends the term “perioperative neurocognitive disorders” to describe both postoperative delirium (POD) and postoperative cognitive dysfunction/decline (POCD), with the latter not having a Diagnostic and Statistical Manual of Mental Disorders 5 (DSM5) criterion for diagnosis [14,15]. Another broad terminology used is “neurological injury post cardiac surgery.” Perioperative stroke, delirium and encephalopathy are some of the neurological sequelae of cardiac surgery [16]. The risk of stroke varies depending on the type of surgery performed [17]. For example, double or triple valve surgery is associated with a 10% risk of stroke, while the risk is lower in patients referred for “beating-heart”, also known as “off-pump”, coronary artery bypass graft (CABG) surgery [17].

In a meta-analysis involving 174,969 patients referred for cardiac surgery, the pooled event rate for early and delayed strokes was each less than 1% [18]. Furthermore, delirium is common in the elderly, with a higher incidence of 12% [19]. There is a paucity of studies reporting neurological complications after cardiac surgery in patients residing in low and middle-income countries (LMIC). In a retrospective chart analysis of 1218 consecutive patients referred for CABG surgery in Johannesburg, the perioperative stroke rate was 1.2% [20]. Moreover, one year after the onset of a stroke, the annual cost in British pounds per person is estimated at GBP 18,081, increasing to GBP 22,961 in patients older than 84 years [21]. These high healthcare costs necessitate an urgent need for strategies that reduce the burden of stroke post cardiac surgery.

## 4. Risk Factors Associated with Neurological Decline

**Figure 1:**
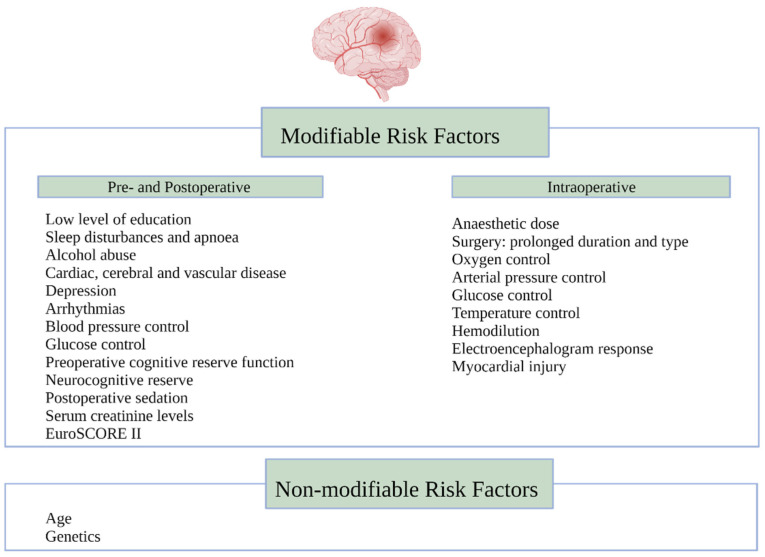
Risk factors for neuronal injury before, during and after cardiac surgery.

In addition to the type of surgery, the stroke risk is increased in patients with existing cerebrovascular disease, peripheral vascular disease, diabetes, hypertension, a history of previous cardiac surgery, preoperative infection, urgent surgery, cardio-pulmonary bypass (CPB) time of more than 2 h, the need for intraoperative hemofiltration, and high transfusion requirements [18]. Risk factors for cognitive decline post cardiac surgery are multifactorial (Figure 1). The neurological outcomes are diverse and have previously been classified as Type I (includes fatal or non-fatal stroke, stupor, or coma at discharge) and Type II, which includes cognitive function deterioration, memory deficit, or seizures [22].

Assessment for cognitive function must be done preoperatively and postoperatively using a validated cognitive test assessment tool. Furthermore, the timing of testing impacts the rate of diagnosing neurological injury post cardiac surgery [23]. Diagnostic tests done within 30 days postoperatively have confounders such as postoperative pain, and medication use may affect the assessment score [15]. Emergence delirium occurs before, during or after awakening from anaesthesia, while postoperative delirium occurs 24–72 h after, and postoperative cognitive decline occurs weeks to months postoperatively [15,24]. Most of the risk factors for POCD are related to their potential for interfering with basic principles of organ protection, such as oxygen delivery, organ perfusion, organ nutritional requirements, pre-existing organ reserves and any toxic exposure during surgery [25]. Prevention of POCD post cardiac surgery is directed at understanding and managing these risk factors.

## 5. Mechanism of Brain Injury

**Figure 2:**
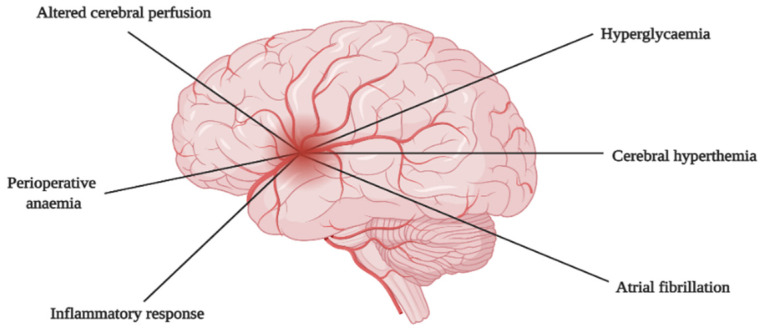
Mechanisms of brain injury.

The mechanism of cardiac surgery-related brain injury is complex and multifactorial. The most common mechanism results from ischaemic causes leading to cerebral hypoperfusion and embolism [26,27]. In addition to ischaemic causes, the inflammatory response, cerebral hyperthermia, hyperglycaemia, perioperative anaemia, and atrial fibrillation have also been implicated (Figure 2) [27].

### 5.1. Altered Cerebral Perfusion

The effects of cardiac surgery and CPB on cerebral perfusion can be classified into cerebral hypoperfusion and reperfusion. Cerebral hypoperfusion resulting from reduced cerebral blood flow (CBF) during CPB is the primary cause of ischaemic brain injury. This form of injury may further be exacerbated by the impaired clearance of micro-emboli during periods of reduced CBF. Advanced age, extensive cerebrovascular disease, and a history of stroke increase the risk of cerebral hypoperfusion [26]. The early period of CPB is usually associated with episodes of a low mean arterial pressure (MAP) during cannula insertions and manipulation of the large vessels of the heart. As a result, cerebral hypoperfusion may occur, and blood flow in the watershed regions of the brain may become critically reduced. Towards the end of CPB, cerebral reperfusion occurs, resulting in the generation of free oxygen radicals [28]. During the ischaemia/reperfusion injury, various secondary mechanisms are initiated and ultimately lead to the death of neurons.

### 5.2. Hypoxia Related Cerebral Injury

Hypoxic conditions occurring during periods of cerebral hypoperfusion result in the upregulation of various molecules, such as hypoxia-inducible factor (HIF) and sulfonylurea receptor 2A (SUR2A) protein, which then initiate events that may lead to neuronal injury [28]. A drop in oxygen levels leads to the translocation of HIF into the neuronal cell nucleus. HIF is comprised of an α and β subunit. The α subunit is constitutively expressed. However, it rapidly degrades under normal oxygen concentrations. Furthermore, hypoxia is associated with a decline in adenosine triphosphate (ATP) levels, which results in the failure of ATP-dependent ion pumps, a rise in intracellular sodium and calcium levels, and ultimately cytoplasmic and mitochondrial swelling, which may lead to cell death [28].

### 5.3. Reperfusion Related Cerebral Injury

The reperfusion phase is associated with reactive oxygen species (ROS) production, predominantly in the mitochondria. The ROS react with nitric oxide (NO) to form peroxynitrite. This molecule is highly reactive and nitrosylates proteins, leading to neuronal functional impairment and cerebral injury [28].

### 5.4. Cerebral Macro- and Micro-Embolism

Cardiac surgery often results in the production of various embolic materials. According to size, emboli can be differentiated into macro-emboli, which occlude flow in arteries with a diameter of 200 μm or more, and micro-emboli, which occlude smaller arteries, arterioles and capillaries. Atherosclerotic plaques arising mainly from the aorta constitute macro-emboli [26], whereas micro-emboli consist of gaseous emboli and biologic aggregates such as thrombus, platelet aggregates and fat [26,28,29]. Emboli arising from inorganic debris such as fragments of polyvinyl chloride tubing and silicone antifoam have been reported in the past [29].

Although the CPB machine has protective mechanisms, such as bubble traps [28], gaseous emboli may still be introduced into the CPB circuit through venous cannulation, administration of drugs and from the open cardiac chambers of the left heart [26]. Furthermore, manipulation of the aorta during surgery increases the risk of embolisation despite the placement of arterial filters [28,30]. Cerebral micro-embolism as a mechanism for cognitive dysfunction is supported by the presence of a relationship between cerebral macro/micro-emboli load during CPB and cognitive dysfunction in various studies [26,31,32,33,34]. Additionally, macro/micro-emboli can also occur during aortic decannulation, particularly when the patient has not received sufficient anticoagulation therapy.

### 5.5. Inflammatory Response

The inflammatory processes associated with CPB further exacerbates neuronal injury [27]. The interaction of patient blood with the CPB components such as tubing, reservoirs, the oxygenator and connections result in the activation of the complement system, leading to the release of pro-inflammatory cytokines such as interleukin (IL)-6, IL-8, IL-10 and tumour necrosis factor-alpha (TNFα) [28,35]. This systemic inflammatory response results in blood-brain barrier leakage, cerebral oedema and ultimately cerebral derangement [28].

### 5.6. Cerebral Hyperthermia

Hyperthermia has also been reported to cause brain damage under various clinical conditions and has been shown to exacerbate neuronal death after ischaemia. The detrimental effects of hyperthermia in the brain are related to hyperthermia-induced cell swelling and necrotic death in the case of very high temperatures [36]. During cardiac surgery on CPB, cerebral hyperthermia might occur due to the proximity of the aortic cannula to the cerebral vessels or underestimating brain temperature by standard monitoring [27].

### 5.7. Hyperglycaemia

The stress response induced by CPB and hypothermia during cardiac surgery leads to elevated serum glucose levels even in non-diabetic patients [27]. The pathophysiology of hyperglycaemia-induced cerebral injury is multifactorial. Hyperglycaemia increases reactive oxygen species production via a protein kinase C-mediated pathway and the increased production of nicotinamide adenine dinucleotide phosphate (NADPH) [37]. The reactive oxygen species can lead to neuronal death, as described earlier. There is also a well-described association between hyperglycaemia and the increased expression of nuclear factor-kappa B [38]. The metabolic effects of hyperglycaemia, such as increased lactic acid production with subsequent acidosis and mitochondrial dysfunction, is another suggested mechanism of hyperglycaemia-associated cerebral injury [39].

## 6. Neuroprotection Interventions

**Figure 3:**
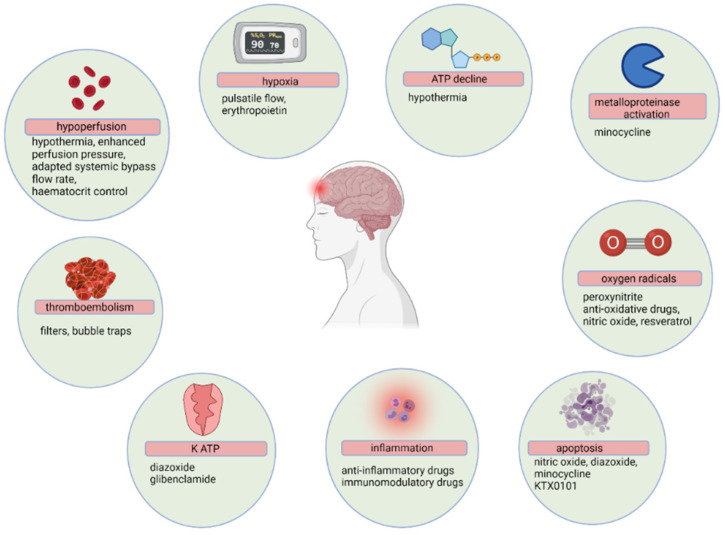
Pathophysiology of the neurological deficit during cardiopulmonary bypass graft surgery. Red bars indicates adverse effects, and green circles reflect proposed neuroprotective strategies.

The quantity and complexity of major cardiac surgeries and procedures is constantly expanding. This population is at risk of postoperative complications, further compounded by physiological and surgical risk factors. Therefore, there is a growing need to mitigate against risk factors associated with neurological injury in the perioperative period. Contemporary data on neuroprotective strategies in children and adults undergoing cardiac surgery are associated with controversies due to multiple contributing factors to brain injury and the assessment of long-term neurocognitive outcomes [40]. Figure 3 demonstrates a schematic representation of the pathophysiology of neurological deficits during CPB, with possible pharmacological and non-pharmacological interventions [28].

### 6.1. Preoperative Strategies

#### 6.1.1. Corticosteroids

The use of corticosteroids is common in the perioperative period due to their effects in downmodulating an inflammatory response, which could decrease the incidence of POCD. However, the complexity of an inflammatory response associated with cardiac surgery and CPB raises doubt that probably not all inflammatory mediators can be suppressed by glucocorticosteroid therapy, even at high dosages. Corticosteroids may also be associated with atrophy and hyperglycaemia, which may exacerbate an ischaemic neurological injury [41]. Large multi-centre RCTs are therefore needed to validate their neuroprotective role.

#### 6.1.2. Erythromycin

Erythromycin induces tolerance against transient global cerebral ischaemia. Postulated mechanisms for neuroprotection in animal models after the administration of erythromycin include the suppression of the immunological response that mediates damage after cerebral ischemia and reprogramming of the transcriptional response to ischaemia [42,43]. Thomaidou et al. found that erythromycin administration attenuated cerebral damage and postoperative cognitive decline after CABG surgery [44]. Since erythromycin has been effectively used in clinical practice with few side effects, these findings place this agent as a promising candidate for potential clinical neuroprotective strategies [45].

#### 6.1.3. Beta-Blockers, Statins and Angiotensin-Converting Enzyme Inhibitors

The neuroprotective benefits of the preoperative administration of beta-blockers and statins in cardiac surgery are not definitive, and further studies are warranted [3,46]. Also, angiotensin-converting enzyme inhibitors (ACEI) are capable neuroprotectants that warrant further investigation in the setting of cardiac surgery on their effects as pre- and postoperative therapy to mitigate against POCD, strokes, ischaemic events, and mortality [47].

#### 6.1.4. Recombinant Human Erythropoietin

Recombinant human erythropoietin (rHuEpo) acts as a pleiotropic tissue-protective cytokine with anti-apoptotic, antioxidant, anti-inflammatory, and neurotrophic factors [48]. It is a protective multipotent tissue factor of the heart and the central and peripheral nervous system that prevents ischemic complications [48]. Preliminary results of a small, single-centre study suggest it could offer neuroprotection during cardiac surgery with an initial dose of 24,000 IU given 24 h before cardiac surgery, a second dose on the day of surgery, and a last dose administered 24 h after surgery [48].

### 6.2. Intraoperative Strategies

#### 6.2.1. Pharmacological

The modern concept of neuroprotective strategies during cardiac surgery is specific, receptor-mediated protective action, i.e., protecting the brain from evolving damage after the initial insult. However, despite impressive short-term protection, most experimental studies have failed to show long-term protection by anaesthetic agents. Therefore, instead of aiming at complete neuroprotection, the current anaesthetic neuroprotection approach should focus predominantly on reducing the severity of the insult.

##### Induction Agents

Barbiturates became less popular when the focus of neuroprotection moved from reduction of energy consumption to receptor-mediated protection, although it is recognised that they can block glutamate receptors, potentiate GABA-ergic activity and inhibit calcium influx, like other anaesthetic agents. These agents are associated with pronounced systemic immunosuppression, increased risk of infection, prolonged effect, and reduced cerebral blood flow [49]. A lack of clinical evidence and complications from extended effects keep barbiturates from being effective neuroprotectants [50].

The mechanism through which propofol causes neuroprotection in humans is not well established. However, suggested mechanisms of action include its role as a sodium channel blocker via excitotoxin mediation, antioxidant properties, and anti-inflammatory or anti-apoptotic mechanisms [51].

##### Inhalational Agents

Volatile anaesthetic agents may provide neuroprotection through reduced excitotoxicity, improved calcium regulation, increased CBF, downregulation of metabolism, reduction of oxidative stress, and increased potassium channel activity [52]. There is no consensus on the type of volatile agent and dose required to produce favourable effects. Animal studies reported reduced neuronal death in the hippocampus of rats when a dose of 1–1.5 minimum alveolar concentration (MAC) of a volatile agent was given 15–30 min before circulatory arrest and at least 0.5 MAC of sevoflurane was given prior to commencement of CPB [49]. A meta-analysis comparing isoflurane and sevoflurane’s neuroprotective capabilities concluded that the choice of a volatile anaesthetic agent does not affect postoperative outcomes in cardiac surgery [53].

Another meta-analysis compared the cerebral-protective effects of volatile anaesthesia and total intravenous anaesthesia (TIVA) in patients undergoing CPB surgery. They found that Mini-Mental State Examination scores were significantly higher in the group who had received volatile agents when compared to propofol, ketamine, and thiopental infusions. No significant differences were found in cerebral metabolism and oxygenation [2]. Experimental data suggests that their preconditioning and postconditioning mechanisms may cause the positive effects of volatile agents. The role of volatiles in pre-and postconditioning (pre-emptive and post-exposure treatment to increase the tolerance of neurons against subsequent lethal insult), reduction of inflammatory markers, improved postoperative neurocognitive function, and CBF is also well recognised [54].

##### Local Anaesthetic Agents

The use of lignocaine in providing neuroprotection has mixed clinical results, especially in transient ischaemia. Its neuroprotective effects are attributed to its ability to cross the blood-brain barrier, block sodium channels, and reduce cerebral inflammation [49]. In addition, lignocaine was shown to increase susceptibility to POCD in diabetic patients [55]. Furthermore, in a meta-analysis involving 688 patients, lignocaine was administered as an infusion from the beginning of the anaesthetic induction to the next 48 h. Results revealed that higher concentrations of lignocaine were associated with more substantial neuroprotective effects [5].

At infusion rates of 1–2 mg/kg/h or 1–2 mg/min, lignocaine increases susceptibility to complications associated with myocardial ischaemia and reperfusion injury. In addition, there is no optimal dosing regimen, and higher dosages of lignocaine may be cardiotoxic. Furthermore, lignocaine has high hepatic extraction, requiring dose reduction in patients with liver disease and reduced cardiac output [56]. Finally, despite conflicting evidence, lignocaine has a promising role as a neuroprotective agent, but further studies are required to investigate the incidence of long-term neurological injury following cardiac surgery.

##### N-Methyl D-Aspartate (NMDA) Receptor Antagonists

Ketamine, an NMDA receptor antagonist, has been investigated due to its potent inhibition of neurotransmission and anti-inflammatory features. It increases absolute regional CBF in a concentration-dependent manner in all brain regions and has a limited incidence of POCD and postoperative delirium [8]. In a multi-centre study of patients undergoing major surgery, ketamine was associated with increased hallucinations and nightmares postoperatively [57]. However, uncertainty still exists regarding its role as a neuroprotectant.

Xenon (Xe) has been shown to have neuroprotective potential due to NMDA receptor blockade. Inhaled Xe (70%) during 60 min of focal cerebral ischaemia in mice improved neurological function and decreased infarct size at 24 h of reperfusion compared with 70% nitrous oxide [49]. While Xe has demonstrated some neuroprotective effects in non-cardiac surgery and animal studies, there is insufficient clinical evidence on potential neuroprotective effects following cardiac surgery.

##### Anti-Inflammatory Agents

Remacidine [50], aprotinin [50,58], complement inhibition by pexelizumab [57], parecoxib, ulinastatin, and minocycline are potential neuroprotectants due to their anti-inflammatory capabilities in animal studies, but whether neurological outcomes are improved in clinical studies has still not been adequately evaluated [59]. Inhibition of inflammation might be helpful, but whether this intervention improves neurological outcomes still needs to be evaluated [59].

##### Alpha-1 Adrenergic Receptor Agonists

Dexmedetomidine has been shown to improve neurological dysfunction in mechanically ventilated patients and in elderly intensive care unit patients after non-cardiac surgery, and has been shown to decrease mortality in septic critically ill patients [60]. It attenuates central sympathetic activity, inhibits inflammation in ischaemic brain tissue via α₂-adrenergic receptor activation [60], protects against neuroapoptosis and has drug-sparing effects through its opioid/GABA-ergic receptors [61]. In addition, animal studies have proven its superior effects when compared to the use of propofol, etomidate, benzodiazepines, and volatile agents in reducing anaesthetic and surgery-induced learning and memory deficits [61]. There is currently an ongoing multicenter RCT investigating the efficacy of dexmedetomidine sedation in reducing the incidence of major postoperative neurocognitive disorders following cardiac surgery [61]. Findings from this trial might strengthen the role of dexmedetomidine as a neuroprotective agent in this population.

While there is some clinical evidence for the role of magnesium sulphate in neuroprotection, the results of RCT studies are contradictory. It is often used due to its role in vasodilatation, stabilising electrochemical gradients, inhibiting glutamate at the NMDA receptor, and its ability to reduce intracellular calcium release [54]. Magnesium has shown effectiveness in improving short-term postoperative memory and cortical control over brain stem function after cardiac surgery [6,62]. Low-dose magnesium, like lignocaine, is a promising neuroprotectant, but clinical evidence of its effectiveness in preventing stroke and ischaemic events in addition to POCD is required. Moreover, studies are required to investigate the optimal magnesium dose required for neuroprotection [6].

##### Osmotic Agents

Osmotic therapy with mannitol was a plausible treatment, as cerebral oedema has been identified as an independent risk factor for postoperative neurocognitive decline. Evidence, however, remains very weak, with few large RCTs performed [51]. Vasodilators such as nitric oxide and chlorpromazine have been suggested as potential drugs with little current clinical support [63].

##### Anti-Oxidative Agents

Diazoxide and glibenclamide bind onto potassium adenosine triphosphate (KATP) receptors to promote ischemic preconditioning. This effect has only been tested on animal models [64,65]. Cyclosporine and resveratrol are anti-oxidative drugs that have been demonstrated to have some neuroprotective effects; however, they have not been used during CPB [66,67].

#### 6.2.2. Non-Pharmacological

##### On-Pump vs. Off-Pump

There has been some debate on the probable advantages of off-pump when compared to on-pump CABG. The Octopus study (2007) investigated long-term cognitive outcomes following cardiac surgery [68]. There was no clinical significance when comparing cognitive decline in both groups after five years following cardiac surgery [68]. The reason for these outcomes is unclear; however, it may result from complex pathophysiological processes that occur during CABG surgery.

##### CPB Duration

The duration of CPB during cardiac surgery has been associated with a variety of adverse outcomes. Longer CPB times are a recognised risk factor for neurological sequelae following cardiac surgery; however, in a meta-analysis by Habibi et al., the use of lignocaine infusions combined with longer CPB times was associated with more favourable neurological outcomes [5]. Perfusion flow generated by CPB is usually non-pulsatile, which contrasts with the physiological pulsatile flow. A paucity of data suggests that pulsatile flow generated by CPB is more beneficial than non-pulsatile CPB [57].

##### Mean Arterial Pressure (MAP)

Clinical studies and literature advise against high mean arterial pressure (MAP) during cardiac surgery. Low MAPs are associated with decreased bleeding and thromboembolic phenomena [69]. In a meta-analysis by Kiabi et al., the neuroprotective effects of low MAP during cardiac surgery were investigated, and the maintenance of MAP below 80 mmHg in this study did not show a decline in the rate of POCD in post-cardiac surgery on CPB [10]. The current anaesthetic practice supports low MAP in decreasing POCD following cardiac surgery, but the evidence-based threshold has not been determined.

##### Degree of Hypothermia

There are comparisons made between the use of moderate hypothermia (20–28 °C) versus deep hypothermia (10–13 °C) [28], or profound hypothermia (<14 °C) against normothermia (36–38 °C) in cardiac surgery on CPB [54]. Hypothermic circulatory arrest (HCA) has several advantages during CPB. It reduces the metabolic rate, free radical production, and post-ischemic cerebral oedema [70]. However, its complications include the risk of coagulopathy, increased CPB time, infections, and increased post-HCA cerebral vascular resistance [70]. Complications arise from prolonged deep hypothermic circulatory arrest (DHCA) and the rewarming process [71]. The most significant disadvantage of deep hypothermia is the process and speed of rewarming, whereby organ dysfunction such as renal failure, coagulopathy, postoperative bleeding, and rhabdomyolysis may occur [28]. Rapid rewarming can be associated with hyperthermia, which aggravates ischaemic brain insults and worsens neurocognitive outcomes [71].

A recent meta-analysis that included 1783 patients found no advantages to DHCA over moderate hypothermic circulatory arrest (MHCA) in elective aortic arch surgery [72]. The alpha-stat pH management strategy was favourable over the pH-stat strategy during CPB in adult patients [73]. The benefits of the former include reduced postoperative acidosis, improved metabolic suppression, and improved cognitive function, although pH-stat continues to be favoured for the paediatric population [54].

##### Cerebral Perfusion

Selective brain perfusion techniques such as antegrade cerebral perfusion (ACP) and retrograde cerebral perfusion (RCP) are utilised during induced hypothermia, and several studies have compared the mode of brain perfusion and temperature management. Clinical evidence favours the use of ACP over RCP [74]. The use of RCP is controversial with potential harm. Leshnower et al. [13] compared DHCA with RCP and MHCA with ACP in an RCT, and clinical outcomes did not demonstrate a difference in neurological fallout when comparing the groups. However, magnetic resonance imaging (MRI) with diffusion-weighted imaging (DWI) revealed a significant number of lesions (100%), consistent with acute cerebral infarction in the MHCA with ACP 7 days post-cardiac surgery, compared to 45% of lesions in the DHCA with RCP group [12]. It is worth noting that this RCT had a small sample size (n = 20), and thus, more RCTs are required. Furthermore, bilateral ACP has an increased arrest time compared to unilateral ACP, but they both have comparable rates of mortality, transient neurological dysfunction (TND), and permanent neurological dysfunction (PND) [75].

Evidence on the use of optimal cannulation during CPB is another topic of debate. There are data on several cannulation techniques with access to major arteries, each with its complication profile [76]. Aortic cannulation and manipulation is one of the standard techniques used during cardiac surgery with CPB. This technique may compromise adequate cerebral blood flow, promote migration of thrombi, and cause complications of haemorrhage and aneurysms [77]. There is evidence that supports the use of periaortic ultrasonography during cardiac surgery. Epiaortic ultrasonography was proven to be superior to manual palpation of atheromatous plaques in the ascending aorta in patients presenting for aortic arch surgery [78]. The presence of these atheromatous plaques is directly related to postoperative strokes [78]. Axillary artery cannulation is also becoming more favourable due to its allowance of a rapid transition to ACP during circulatory arrest [77].

##### CPB Circuit

The inflammatory response associated with the CPB circuit may also be reduced by using leucocyte filters, heparin-coated circuits, and tubes coated with specific polymers [79]. This inflammatory reaction usually activates endothelial cells, leukocytes, platelets, complement factors, and coagulation [79]. Gasparovic et al. investigated the use of remote ischaemic preconditioning (RIPC) in patients undergoing elective CABG [13]. When compared to standard treatment, there were no significant structural, functional, and neurocognitive decline differences.

##### Glycaemic Control

Hyperglycaemia in hospitalised patients is often defined as serum glucose levels of more than 7.8 to 11 mmol/L (140–180 mg/dL) [80]. The surgical, anaesthetic, and CPB-related stress responses, together with the use of glucose-containing fluids, may increase plasma glucose levels during cardiac surgery [81,82]. The mechanisms causing neurological effects involve an increased lactate production substrate, more release of glutamate and aspartate (key mediator in the ischaemic cascade), and general enhancement of the inflammatory process [83]. It is therefore prudent to avoid hyperglycaemia in the perioperative period.

##### Neuromonitoring

Neuromonitoring techniques are valuable in both the intra- and postoperative periods. The use of processed electroencephalogram (EEG) or bispectral (BIS) monitoring, cerebral oximetry (near-infrared spectroscopy—NIRS), and transcranial doppler ultrasound, alongside other physiological monitors, should be used routinely to detect and prevent damage resulting from ischaemic hypoxaemia, embolism, hypocapnia, arterial hypotension, low cardiac output and variations in temperature during cardiac surgery and in the postoperative period. Available data suggest that these technologies identify adverse neurologic events and may even predict outcomes [40]. However, postoperative neuroprotective strategies that mitigate cerebral injury following cardiac surgeries are not widely studied.

## 7. Assessing the Cause, Extent and Impact of Neuronal Injury

Brain imaging plays a role in determining the location, size and extent of injury, providing useful information that guides short-term and long-term therapeutic interventions. Patients with clinical features suggestive of neuronal injury such as cognitive decline, seizures, or an altered state of consciousness after cardiac surgery should ideally be referred for brain imaging.

### 7.1. Computed Tomography

Computed tomography (CT) has established itself as the primary imaging modality for brain imaging owing to its availability, speed of obtaining images and accurate delineation of parenchymal changes. Although studies comparing outcomes in patients referred for CT brain vs. other modalities are lacking, most trials have demonstrated that CT brain imaging is crucial before therapeutic interventions, particularly before administering thrombolytic therapy such as tissue plasminogen activating factor [84]. Moreover, multimodal imaging, comprising enhanced CT, CT angiogram of the extracranial and intracranial neck vessels and CT perfusion, has a higher sensitivity of 79% in detecting infarction, ischemia, and culprit lesions in stroke patients [85]. Although the sensitivity of combined techniques is higher than that of each modality, the higher radiation dose is a significant concern.

#### 7.1.1. Non-enhanced Computed Tomography

Non-enhanced CT should be performed urgently in patients with clinical manifestations suggestive of acute stroke to exclude intracranial haemorrhage and identify large (>100 mL or more than one-third of a brain territory at risk), well-established infarcts [86]. Early signs of ischemic brain injury or arterial occlusion include the hyperdense middle cerebral artery sign, indicative of a thrombus or embolus in the first portion of the middle cerebral artery [86]. In addition, the loss of the gray-white differentiation in the cortical ribbon, particularly at the lateral margins of the insula or the lentiform nucleus, and sulcal effacement may be evident. These signs are poor prognostic factors and may be detected within 6 h of the onset of symptoms in up to 82% of patients with ischemia in the middle cerebral artery territory [86].

#### 7.1.2. Computed Tomography Angiography (CTA)

After administering a bolus of intravenous contrast, culprit lesions in the microvasculature from the aortic arch to the cranial vessels are identified within 15 s, evaluated and treated depending on the degree of stenosis. Multiphase CTA tracks the bolus beyond the arterial phase and has several advantages over single CTA techniques, including improved culprit lesion detection and collateral blood supply assessment [87].

#### 7.1.3. Computed Tomography Perfusion

With this imaging modality, serial images of brain tissue volume are acquired over time during intravenous contrast injection, creating a time-density plot. Thus, parameters such as the cerebral blood volume and mean transit time can accurately measure the penumbra, defined as an ischemic but viable tissue [88].

### 7.2. Magnetic Resonance Imaging

Brain magnetic resonance imaging detects small cortical or subcortical lesions more easily than CT in the acute phase. Diffusion-weighted magnetic resonance imaging identifies ischemic lesions with a sensitivity rate above 88% and 95% specificity [89]. Ischaemic lesions appear as hyperintense areas and correlative hypointense areas on apparent diffusion coefficient maps within 3 min of stroke onset [89]. Other imaging findings predictive of good 3-month outcomes on CT brain and MR imaging include infarct confinement in the gray matter, corticospinal tract sparing and a scattered infarct structure [90]. Like CT angiography, MR angiography identifies intravascular occlusions and evaluates the carotid bifurcation in acute stroke patients.

### 7.3. Functional Nuclear Imaging

Brain perfusion is assessed after the intravenous administration of lipophilic radiopharmaceuticals that cross the blood-brain barrier. The distribution of the radiopharmaceutical in the acquired brain images typically reflects neuronal activity. Positron emission tomography evaluates blood flow or oxygen extraction. Prior to the scan, patients should avoid excessive stimulants such as noise and light as these stimulants may be registered as brain activity, falsely increasing blood flow and subsequent tracer delivery, masking underlying ischemic or infarcted tissue.

#### 7.3.1. Single-Photon Emission Computed Tomography

This non-invasive imaging technique measures regional cerebral haemodynamics, dopamine and other neurotransporter distribution. SPECT assessment can confirm brain death, depicted by decreased or absent cerebral blood flow [91].

#### 7.3.2. Positron Emission Tomography

Inert and freely diffusible radiopharmaceuticals are used to assess cerebral blood flow. During the scan, radiolabeled oxygen activity in the arterial blood is recorded, and local cerebral blood flow values are calculated. Brain perfusion studies with PET are not routinely performed due to the higher cost of housing a cyclotron on site, required for manufacturing perfusion radiopharmaceuticals [91].

## 8. Neuroprotection Recommendations

The lack of standardised protocols for neuroprotective strategies in patients undergoing cardiac surgery is related to the scarcity of robust evidence in support of perioperative interventions to prevent neurological fallout. Nevertheless, poor postoperative neurological outcomes carry a disease burden in this patient population. A variety of approaches exists among cardiac anaesthesiologists and perfusionists worldwide in assessing and preventing neurological injury during cardiac surgery. Table 2 represents evidence-based recommendations of preventative strategies in the perioperative period.

## 9. Future Directions

Targeted therapies and emerging imaging technology will help to reduce the incidence of neurological fallout. Multidisciplinary, translational research is the cornerstone to identifying and reducing neurological injury in patients undergoing cardiac surgery. We recommend multidisciplinary collaborative efforts between anaesthesiologists, cardiologists, cardiothoracic surgeons, and neurologists when managing patients referred for cardiac surgery. Future studies should focus on applying techniques that assess complex relationships between clinical and imaging variables.

## 10. Conclusions

The overall evidence for pharmacological neuroprotection is weak. Most neuroprotective strategies are based on animal studies, which cannot be fully extrapolated to the human population. Furthermore, there is still no universal consensus on the optimal neuroprotective strategies for patients undergoing cardiac surgery. Large multicenter RCTs are still required to evaluate the beneficial effects of different neuroprotective techniques.

## Figures and Tables

**Table 1:** Summary of randomised control trials comparing interventions to standard therapy for neuroprotection in cardiac surgery.

Author & Publication Year	Intervention	Placebo/Standard Care	Outcome	Results
Habibi et al. [5], 2018	Lidocaine* n = 340	Normal saline infusion *n* = 348	Development of POCD (decline of >1 SD postoperatively when compared to preoperative baseline) - Wechsler Adult Intelligence Scale (WAIS)	POCD: 29% for the lidocaine group and 39% for the control. Lidocaine use reduced POCD. Better outcomes were in younger patients, male gender, more prolonged CPB, and higher concentrations of lidocaine.
Pearce et al. [6], 2017	Magnesium* n = 582	Normal saline *n =* 582	Functional neurological assessment - Western Perioperative Neurologic Scale (WPNS)	The measurement of neurological outcomes varied in the different studies. Use of different doses at induction of anaesthesia, up to 48 h post-surgery. Methodological heterogeneity existed in these studies. There were improved functional neurological outcomes in the magnesium group for patients with global ischaemia, but magnesium does not improve long-term cognitive function.
			Neurophysiological assessment - Cognitive P300 Visual Evoked Potential - Neuron-Specific Enolase (NSE) - Mini-mental State Exam Forward and Backward Digital Span Tests	
			Neuropsychological assessment - Hopkins Verbal Learning Test (HVLT) - Controlled oral word association test (COWAT) - Boston Naming Test (BNT) - Short Story module of the Randt Memory Test - Wechsler Adult Intelligence Scale Revised (WAIS-R) Test	
Chen et al. [2], 2017	Inhalational/Volatile agents - Desflurane - Sevoflurane - Isoflurane *n* = 272	TIVA techniques - Propofol - Thiopental - Ketamine - Midazolam *n* = 277	Serum protein S100B levels were the primary outcome measure.	Levels of S100B protein were lower in the Inhalational group when compared to the TIVA group. MMSE scores were higher in the inhalational agents’ group. No significance between inhalational and TIVA group in SjVO_2_, D (a-v)O_2_ and O_2_ER
			Secondary outcomes: - Mini-mental State Examination (MMSE) - Jugular bulb venous oxygen saturation (SjVO2) - Arterio-venous oxygen content (D (a-v)O2 - Cerebral oxygen extraction ratio (O2ER)	
Momeni et al. [7], 2021	Propofol plus dexmedetomidine infusion *n* = 204	Propofol plus standard saline infusion *n* = 207	Incidence of POD: - Richmond Agitation Sedation scale (RASS) - Confusion Assessment Method for Intensive Care Unit (CAM-ICU)	POD occurred in 18% of dexmedetomidine and in 19% of the placebo group. These results did not show any statistical significance.
Hudetz et al. [8], 2009	Ketamine bolus at induction *n* = 26	Normal saline *n* = 26 Non-surgical group *n* = 26	Neurocognitive and neurological testing: - Verbal recent memory - Nonverbal recent memory - Executive functions	A decrease in cognitive function by at least 2 standard deviations in the placebo group vs. ketamine group when compared to non-surgical controls was observed.
			Depression: - Geriatric depression scale	
			Vascular dementia: - Hachinski Ischaemia scale	
Gamberini et al. [9], 2009	Rivastigmine* n = 57	Placebo *n* = 56	Delirium screening: - Confusion Assessment Method (CAM)	There was no difference between the rivastigmine and placebo groups. Delirium occurred in 17 patients (30%) of the placebo group and 18 patients (32%) of the rivastigmine group.
			Cognitive assessment: - Mini-Mental State Examination (MMSE) - Clock drawing test (CDT)	
Kiabi et al. [10], 2019	MAP < 80 mmHg	MAP 80 ≥ mmHg	Incidence of POCD: - Wechsler Adult Intelligence Scale (WAIS-R) - Trail making A and B - Grooved pegboard test (pegs) - Boston Naming, Benton Visual Retention and Recognition Test - Controlled Oral Word Association - Mattis–Kovner Verbal Recall and Recognition and finger tapping test - Ammons Quick Test - Control tests: CES-D and SF-36 health survey - Mini-Mental State Examination (MMSE)	POCD occurred in 6.4% of all cases. The maintenance of low MAP was not associated with a decline in POCD. A shorter duration of CPB was associated with a reduction in POCD regardless of the groups (lower MAP vs. standard of care).
Sedrakyan et al. [11], 2006	Off-pump CABG	On-pump CABG	Postoperative strokes	Off-pump was associated with the least occurrences of strokes.
Leshnower et al. [12], 2019	Deep hypothermic circulatory arrest (DHCA) with retrograde cerebral perfusion (RCP) * n = 11	Moderate hypothermic circulatory arrest (MHCA) with antegrade cerebral perfusion (ACP) -cooled to nasopharyngeal temperatures of 20 °C to 28 °C *n* = 9	Neurological examination and classification: - Stroke (deficit > 24 h) - TIA (reversible deficit < 24 h) - TND (transient neurological dysfunction)	There was no clinically significant neurological injury present in both groups. Clinical parameters and S-100 levels were very similar; however, MRI DWI lesions that are consistent with acute cerebral infarction were present in 45% of the DHCA + RCP compared to 100% of MHCA + ACP
			National Institute of Health stroke scale (NIHSS) - 42-point scale	
			Serum levels of S-100 MRI examination with diffusion-weighted imaging (DWI)	
Gasparovic et al. [13], 2019	Remote ischaemic preconditioning (RIPC)* n = 33	Standard care *n* = 33	Structural and functional cerebral changes: - MRI on a 3T resonance magnetic scanner	No statistically significant difference between the two groups. New brain ischaemia occurred in 9 patients (27%) of the RIPC group versus eight patients (24%) of the control group.
			Neurocognitive testing: - Montreal cognitive assessment (MOCA) - Trail making tests A and B (TMT-A/TMT-B)	
Uysal et al. [4], 2020	Cerebral oxygenation monitoring* n = 59	Control group: Cerebral oxygenation information was hidden from investigators No intervention protocol *n* = 66	Cognitive testing: Cognitive Stability Index HeadMinder battery (HeadMinder, Inc, New York, NY, USA) - Response speed - Processing speed - Attention - Memory	No significant differences in cognition existed between the intervention and control group at T2. The mean memory score at T3 was better in the intervention group.

Abbreviations: RCT, randomised control trial; CPB, cardiopulmonary bypass; POCD, postoperative cognitive dysfunction; MM-RR, Mantel–Haenszel risk ratio; CI, confidence intervals; OR, odds ratio; TIVA, total intravenous anaesthesia; WMD, weighted mean difference; POD, postoperative delirium; CABG, coronary artery bypass grafting; MAP, mean arterial pressure; SD, standard deviation. Lidocaine* bolus of 1–1.5 mg/kg. Infusion of 2–4 mg/kg/hour: dependent on centres’ protocol. Lidocaine plasma concentrations ranged between 7 and 30 µmol/L. Magnesium* doses of at least 2 g within 24 h of cardiac arrest or cardiac surgery. Rivastigmine*, 1.5 mg dose 8 hourly, from evening preoperatively; a total of 22 doses (up to day 6 postoperatively). Deep hypothermic circulatory arrest (DHCA) with retrograde cerebral perfusion (RCP)*, cooled to nasopharyngeal temperatures of 14.1 °C to 20 °C. Remote ischaemic preconditioning (RIPC)* by the use of BP cuff in the upper limb with three alternating cycles of inflation (200 mmHg for 5 min) and deflation for 5 min (reperfusion). Cerebral oxygenation monitoring*: Cerebral deoxygenation episodes < 60% for > 60 s.

**Table 2:** Neuroprotection strategies for patients undergoing cardiac surgery.

**Preoperative Strategies**	Identifying high-risk patients Preoperative cognitive screening in high-risk patients Multidisciplinary consultation (anaesthesiologists, cardiologists, neurophysiologists, neuropsychologists and surgeons) Prehabilitation Planning on individualized surgical and anaesthetic techniques	
**Intraoperative Strategies**	**Non-pharmacological** Minimally invasive surgical techniques where possible (OPCAB, TAVI, MIMVS) Maintaining MAP above 60 mmHg Adequate anticoagulation Use of epi-aortic ultrasound during cannulation Maintenance of adequate pump flow rates Monitoring cerebral oximetry (INVOS) within 20% of baseline values Monitoring depth of anaesthesia and cerebral metabolism with BIS values ~45 Maintenance of Hct > 25% pH stat when cooling < 28 °C and alpha-stat when not cooling < 28 °C Maintain PCO_2_ between 35–45 mmHg Titrate FiO_2_ to avoid hyperoxia Maintain normoglycaemia Maintain hypothermia for brain protection, including use of an ice hat during surgery that necessitates deep hypothermic arrest Head down position during decannulation Use of TEE to exclude air emboli post-surgery	**Pharmacological** Volatile anaesthetic agents TIVA techniques MgSO_4_ at 7 mg/kg Lignocaine Steroids Dexmedetomidine Ketamine
**Postoperative Strategies**	Avoid hypoxaemia Early extubation and prevention of respiratory tract infections Management of co-morbid factors Glucose control Blood pressure control Optimise lipid therapy Early enteral feeding Early mobilization	

MAP, mean arterial pressure; OPCAB, off-pump coronary artery bypass; TAVI, transcatheter aortic valve implantation; TEE, trans-oesophageal echocardiography; INVOS, brain oxygenation saturation monitor; TIVA, total intravenous anaesthesia; BIS, bispectral index; MIMVS, minimally invasive mitral valve surgery; Hct, haematocrit; PaCO_2_, partial pressures of carbon dioxide; FiO_2_, fraction of inspired oxygen; PaO_2_, partial pressures of oxygen; MgSO_4_, magnesium sulphate.

## Data Availability

Not applicable.
